# Exploring research participants’ perceptions and comprehension of the informed consent process in a pre-exposure HIV prevention study: a case study

**DOI:** 10.1186/1742-4690-9-S2-P238

**Published:** 2012-09-13

**Authors:** S Ruzario, W Mavhu, R Musesenngwa, R Zinyama-Gutsire, R Gunda, M Phiri, T Rossouw

**Affiliations:** 1 Medical Research Council of Zimbabwe, Harare, Zimbabwe; 2 ZAPP University of Zimbabwe, Harare, Zimbabwe; 3 University of Pretoria, Pretoria, South Africa

## Background

Ensuring informed consent is a complicated component of clinical trials particularly with HIV prevention trials conducted in resource-limited settings. An inherent challenge of the informed consent process for HIV prevention studies is making sure trial participants understand that their participation does not increase exposure to HIV. Participants need to comprehend that partaking in such trials does not necessarily protect them from HIV. It is important to continuously monitor the informed consent process for clinical trials with view to improving the procedure.

## Methods

Between June and September 2011, gender-specific in-depth interviews were held with interviewees who had been purposively selected from female participants who had exited a vaginal HIV prevention study. An interview guide was used to elicit views around the informed consent process. Discussions were conducted and audio-recorded. Audio-recorded data were transcribed, translated verbatim into English, coded using NVivo 8 and analysed using grounded theory principles.

## Results

Twenty interviewees were held. Key information about the study was given as participants articulated study aims well. The informed consent process had been rushed and participants had not had enough time to decide and consult. Due to both excitement and anxiety, participants felt pressured to sign consent forms before comprehending some aspects of the study. Some found it difficult to ask questions. Data suggested that both the study procedure and duration had not been fully explained. Mixed feelings on male partner involvement in decision-making around study participation existed, with some feeling that spouses should have been involved and others stating that partner consultation did not matter.

## Conclusion

This study elicited some of the issues that characterise the informed consent process for clinical trials conducted in resource-limited settings. It highlighted the need for researchers’ ingenuity in order to come up with strategies that tailor the informed process to suit specific needs and circumstances of individual participants.

